# Immunological and Clinical Characteristics of Systemic Lupus Erythematosus: A Series from Morocco

**DOI:** 10.1155/2018/3139404

**Published:** 2018-09-30

**Authors:** Zeineb Zian, Mouna Maamar, Mohamed El Aouni, Amina Barakat, Naima Ghailani Nourouti, Rajae El Aouad, Naima Arji, Mohcine Bennani Mechita

**Affiliations:** ^1^Biomedical Genomics and Oncogenetics Research Laboratory, Faculty of Sciences and Techniques of Tangier, University Abdelmalek Essaâdi, Tetouan, Morocco; ^2^Internal Medicine Department, Hospital Ibn Sina, Rabat, Morocco; ^3^Autoimmunity Laboratory, National Institute of Hygiene, Rabat, Morocco

## Abstract

Systemic Lupus Erythematosus (SLE) is a complex autoimmune disease with a high female predominance. To date, studies about SLE in Morocco are few. This retrospective study describes the clinical and immunological features in a series of 50 SLE Moroccan patients in University Hospital Center of Rabat, Morocco, between December 2011 and December 2013. All patients were screened for antinuclear antibodies (ANA) and anti-DNA antibodies by indirect immunofluorescence, followed by identification of anti-extractable nuclear antigen antibodies by ELISA. The female to male ratio was 6.1:1. Mean age was 31.72 years. The main clinical manifestations were arthritis (82%), mucocutaneous manifestations (80%), renal manifestations (50%), and hematological features (46%). Of the mucocutaneous features, the highest frequencies were observed in the malar rash (68%) and photosensitivity (60%). Of the hematological features, lymphopenia was most frequently observed in 30% of patients, followed by hemolytic anemia in 16% and leucopenia and thrombocytopenia in 8%. Central nervous system was involved in 10%. ANA were found in 88%, anti-DNA antibodies in 56%, and anti-Sm antibodies in 50%. Anti-SSA, anti-SSB, anti-Sm/RNP, and anti-Scl70 antibodies were detected in 38%, 10%, 48%, and 8%, respectively. Our data show that, in our patients, the main clinical and immunological features of SLE remain comparable to patients from other Arab countries.

## 1. Introduction

Systemic Lupus Erythematosus (SLE) is a complex autoimmune disease with a worldwide distribution and an unknown etiology [1]. It is characterized by a great clinical polymorphism and female predominance [2, 3]. The appearance, progression, and outcome of SLE are influenced by genetic, immunological, and environmental factors [4, 5]. Ethnicity also seems to contribute to the expression and heterogeneity of the clinical and immunological features of disease [6]. To date, only scarce data about SLE in the Moroccan population is available [7, 8]. The aim of this study was to describe the clinical and immunological profile of Moroccan patients diagnosed with SLE.

## 2. Patients and Methods

### 2.1. Patients

It is a retrospective study including 50 SLE patients (43 females and 7 males) diagnosed in the Internal Medicine Department at the University Hospital Center Ibn Sina (Rabat, Morocco) during the period from December 2011 to December 2013. This Hospital provides a wide geographical coverage of the country in enrolling such patients. All patients satisfied at least four of the American College of Rheumatology (ACR) criteria for the classification of SLE [4, 9].

The study was approved by Ethics Committee for Biomedical Research in the Faculty of Medicine and Pharmacy of Rabat (CERB), Morocco. All involved patients signed written informed consent.

### 2.2. Immunological Tests

Tests for ANA and anti-DNA antibodies were performed by indirect immunofluorescence assay (IFA), using HEp-2 cells (Bio-Rad Laboratories, CA) as substrate for ANA and* Crithidia luciliae* (Bio-Rad Laboratories, CA) for anti-DNA. The clinically significant titers for ANA and anti-DNA were 1:160 and 1:5, respectively. Autoantibodies to extractable nuclear antigens (ENA) (Sm, SSA, SSB, Sm/RNP, and Scl70) were studied by ELISA (ANA-6 Profile kit, Bio-Rad Laboratories). Values greater than 25 Enzymatic Units (EU) were considered positive. The IFA and ELISA tests were performed on an automated system (Biorad PhD™ system). Analysis of all sera was conducted at the Autoimmunity Laboratory of the National Institute of Hygiene (Rabat, Morocco).

Statistical analyses were performed using Chi-square test with Yates's correction or Fisher's exact test wherever appropriate to assess significance difference in frequencies between clinical and immunological findings seen in our study and some Middle East and North African (MENA) countries. A two-sided P value < 0.05 was considered statistically significant. All statistical comparisons were performed using COMPARE2 software (version 1.02).

## 3. Results

### 3.1. Clinical Manifestations

Fifty patients fulfilling at least four of the ACR criteria for SLE were enrolled in this study during the period from December 2011 to December 2013. There were 43 females (86%) and 7 males (14%) with the female: male ratio being 6.1:1. The mean age was 31.72 ± 14.36 (mean ± SD) ranging between 12 and 86 years.

In this study, the diagnosis of SLE was reached based on clinical and serological features of the disease according to the classification criteria of ACR, by an expert clinician, after excluding differential diagnoses. Patients who presented clinical signs of SLE but have ANA negative, for whom the diagnosis of SLE was admitted by their internist doctor after elimination of differential diagnoses, were also included. These were six patients with the most common manifestations at diagnosis being hematological disorders (hemolytic anemia, lymphopenia, and leucopenia), arthritis, renal complications, and mucocutaneous manifestations (malar rash and photosensitivity). From those with ANA negative, one woman aged 86 years was diagnosed as elderly SLE. The diagnosis was based on 4 clinical criteria of the ACR SLE classification that she fulfilled at presentation at the hospital (arthritis, hemolytic anemia, thrombocytopenia, and renal complications).

The frequency of different clinical manifestations is shown in Table 1. Articular manifestations occurred in 41 patients (82%), of whom 11 (22%) presented nonerosive arthritis. Mucocutaneous manifestations were the most frequent clinical features and they were found in 40 patients (80%). Malar rash was mostly presented with a frequency of 68% (34 cases), followed by photosensitivity in 30 cases (60%) and oral ulcers in 4 cases (8%). Renal involvement was presented in 25 (50%) patients. Hematological manifestations were seen in 23 (46%) patients of our group; lymphopenia was the most common hematological abnormality and it was found in 15 patients (30%). Hemolytic anemia occurred in 8 cases (16%). Leucopenia and thrombocytopenia were observed in 4 cases (8%). Five out of fifty (10%) patients had neurological features. Cardiac manifestations were recorded in 4 (8%) patients. Arterial hypertension was found in 2 (4%) patients and ocular involvement was only observed in 1 (2%) patient.

### 3.2. Immunological Manifestations

The frequency of the main autoantibodies detected in our SLE patients is presented in Table 2. Of the total of fifty patients studied, 44 (88%) patients had positive ANA. Anti-DNA antibodies were seen in 28 (56%) patients. Anti-Sm antibodies were found in 25 (50%) patients. Anti-SSA antibodies were positive in 19 (38%) and anti-SSB in 5 (10%) patients. Twenty-four (48%) patients had anti-Sm/RNP and 4 (8%) anti-Scl70 antibodies.

## 4. Discussion

In this work, we describe the main clinical and immunological features in 50 Moroccan SLE patients diagnosed in the University Hospital Center Ibn Sina, Rabat. Our retrospective study includes urban Moroccan patients issued from Arab and/or Berber population groups and shared globally similar socioeconomic conditions and lifestyle.

In accordance with literature data, women were more affected than men in our series, but the female to male ratio differs according to published studies [6, 10–15]. Among the most frequently observed manifestations in our series, arthritis was found in 82%, which was comparable to that observed in other studies (Table 3). Mucocutaneous manifestations were marked by a high frequency of malar rash and photosensitivity. These findings were in agreement with data reported in previous studies in particular in the Tunisian report [12]. Photosensitivity frequency observed in our patients was significantly higher compared to Saudi study (p < 0.001) (Table 3). This may be in part explained by the level exposure of our patients to sunlight compared to Saudi traditional practices protecting their faces against sun.

Lymphopenia represents the commonest hematological abnormalities occurred in our patients (30%). A likely correlation between lymphopenia prevalence and disease activity has been shown [16]. As our patient's group was not followed up, correlation between both of these parameters could not be demonstrated. Hemolytic anemia was shown to be significantly lower in our series than that reported in Saudi Arabian SLE patients (16% versus 63.1%, p < 0.001) and higher than Tunisians but slightly significant (16% versus 6%, p = 0.05) (Table 3). Generally, the difference observed in hematological abnormalities frequency between studies is mainly due to the difference in the frequency of both leucopenia and lymphopenia [16–18].

Almost half of SLE patients develop renal disease [19], and this has a direct impact on morbidity and mortality [20]. Renal manifestations frequency in our series was similar with all compared Arab studies (Table 3). Neurological manifestations were less frequent than other clinical characteristics [6, 10, 12], but their frequency (10%) in our report was significantly lower compared to Omani (33.8%) and Saudi Arabian (27.6%) studies (p < 0.05). These manifestations have been detected in SLE patients who take inadequate or wrong treatment [21]. The different prevalence in these clinical features might be attributed to the geographic variation and environmental, cultural, and socioeconomic factors, which could play an important role in the expression of the disease [22].

ANA frequency in our patients (88%) was significantly lower compared to that reported in other Arab studies (p < 0.05) (Table 3). Similarly to the Tunisian series, anti-DNA antibodies were positive in more than half of patients (56%), but their frequency was significantly higher in other series (p < 0.001) (Table 3). This could be explained by the size limited of our series, differences in test kits used, and the cut-off levels in each population. Half of our patients had anti-Sm antibodies, which is particularly elevated in our series. Such higher frequency has been already described in North Africa [12]. Conversely, lower proportions have been observed in patients from the Middle East [6] and Spain [15]. Frequency of these antibodies varies between patients depending on the ethnic origin [23]. Anti-SSB antibodies are particularly frequent in Sjögren's syndrome. Their presence in SLE patients could be an indicator of development of this syndrome [24], hence their lower frequency observed in our study (10%). This difference in antibody frequencies between studies could be explained by the variability of sensitivity related to the methods used in antibody detection and the ethnic origin. Anti-Scl70 antibodies are specific to diffuse form of Systemic Sclerosis (SSc) disease [25] and rarely associated with SLE [26, 27], which could explain the lower frequency found in our report (8%). Their presence in SLE patients may be of a great value in studying the disease progression since a correlation between the presence of anti-Scl70 antibodies in SLE patients and the activity of the disease has been suggested [28, 29]. The similarity in some manifestations of the SLE observed between our data and the Tunisian study might be related somewhat to the shared North African origin as well as the same Mediterranean weather, particularly the high level of sunshine in these countries. It could also be explained by the same socioeconomic factors of both populations studied.

Our study showed some limitations. The retrospective character did not allow us to have much information that could be of great interest (follow-up of patients, disease duration, and the use of medications). Socioeconomic factors and the fact that this pathology is not widely known by Moroccans probably influenced the size of the population studied.

Since the number of patients studied is limited, we could not draw firm conclusions about the characteristics of SLE in Moroccan patients. To a better understanding of the SLE features in Morocco, larger and multicenter studies are needed. Epidemiological studies concerning SLE incidence, prevalence, influence of environmental, and genetic and ethnicity factors are also warranted as there is no epidemiologic data for this disease in the national scale.

## 5. Conclusion

Our results showed that the main clinical and immunological features remain comparable to other Arab studies. We described high frequencies in arthritis, mucocutaneous manifestations, renal disorders, and hematological abnormalities in our SLE patients. The ANA and anti-DNA antibodies were more prevalent compared to other immunological factors in our series, consistent with literature data. Multicenter prospective studies with larger population are needed to confirm our findings.

## Figures and Tables

**Table 1:** Clinical manifestations of 50 SLE patients.

**Manifestations**	**No**	**Frequency**
Mucocutaneous	40	80%
Malar rash	34	68%
Photosensitivity	30	60%
Oral ulcers	4	8%
Ocular involvement	1	2%
Hematological	23	46%
Hemolytic anemia	8	16%
Leucopenia	4	8%
Lymphopenia	15	30%
Thrombocytopenia	4	8%
Arthritis	41	82%
Renal involvement	25	50%
Cardiac	4	8%
Neurological	5	10%
Arterial hypertension	2	4%

**Table 2:** The main autoantibodies in 50 SLE patients.

**Immunological tests**	**No**	**Frequency**
ANA	44	88%
Anti-DNA	28	56%
Anti-Sm	25	50%
Anti-SSA	19	38%
Anti-SSB	5	10%
Anti-Sm/RNP	24	48%
Anti-Scl70	4	8%

**Table 3:** Comparison of the main clinical and immunological features of SLE patients from our series and some countries of MENA region.

Manifestations	Our study 2018	Dubai 2008 [6]	Saudi Arabia 2009 [10]	Oman 2003 [11]	Tunisia 2003 [12]
Number of patients	50	151	624	73	100
Mean age (years)	31.7	35.5	34.3	-	32
F:M ratio	6.1:1	20.5:1	9.8:1	24:1	11.5:1
Mucocutaneous (%)	80	78.1	64.3^a^	-	-
Malar rash (%)	68	60.3	47.9^a^	-	63
Photosensitivity (%)	60	43	30.6^b^	-	53
Hematological (%)	46	61.6	78^b^	-	-
Hemolytic anemia (%)	16	9.9	63.1^b^	-	6
Leucopenia (%)	8	53.6	30.1^b^	23.5	-
Lymphopenia (%)	30	-	40.3	49^a^	50^a^
Thrombocytopenia (%)	8	18.5	10.9	10.4	12
Arthritis (%)	82	88.1	80.4	47.8^b^	78
Renal involvement (%)	50	51	47.9	50.7	43
Neurological involvement (%)	10	15.9	27.6^a^	33.8^a^	25
ANA (%)	88	98^a^	99.7^b^	97^a^	100^b^
Anti-DNA (%)	56	88.7^b^	80.1^b^	92^b^	56
Anti-Sm (%)	50	19.7^b^	41.6	50	61.2
Anti-SSA (%)	38	52.3	53.1^a^	44	64^a^
Anti-SSB (%)	10	19.8	26.1^a^	41^**b**^	33.6^a^

^a^ *P* value < 0.05, compared with our data.

^b^ *P* value < 0.001, compared with our data.

- means not available.

## Data Availability

The datasets analyzed during the current study will be available from the corresponding author on reasonable request.
